# Circulating Expression Level of LncRNA Malat1 in Diabetic Kidney Disease Patients and Its Clinical Significance

**DOI:** 10.1155/2020/4729019

**Published:** 2020-08-01

**Authors:** Lian-ji Zhou, Da-wei Yang, Li-Na Ou, Xing-Rong Guo, Biao-liang Wu

**Affiliations:** ^1^Jinan University, Guangzhou 510632, China; ^2^Department of Endocrinology, The Affiliated Hospital of Youjiang Medical University for Nationalities, Baise, Guangxi, China 533000

## Abstract

**Background:**

Long noncoding RNA MALAT1 is closely related to diabetes and kidney diseases and is expected to be a new target for the diagnosis and treatment of diabetic nephropathy.

**Objective:**

This study aimed to explore the circulating expression level and significance of lncRNA Malat1 in patients with type 2 diabetes mellitus (T2DM) and diabetic kidney disease (DKD).

**Methods:**

Quantitative real-time PCR (qPCR) was conducted to assess the expression of lncRNA Malat1 in 20 T2DM patients, 27 DKD patients, and 14 healthy controls, and then, the clinical significance was analyzed.

**Results:**

LncRNA MALAT1 expression in peripheral blood mononuclear cells (PBMC) was significantly upregulated in T2DM and DKD groups when compared to control. Pearson's correlation analysis showed correlation of lncRNA MALAT1 levels with ACR, urine *β*2-microglobulin (*β*2-MG), urine *α*1-microglobulin (*α*1-MG), creatinine (Cr), and glycosylated hemoglobin (HbA1c), while negative with superoxide dismutase (SOD) (*r* = −0.388, *P* < 0.05). Binary regression analysis showed that ACR, creatinine, *α*1-MG, and LncRNA Malat1 were the risk factors for diabetic nephropathy with OR value of 1.166, 1.031, 1.031, and 2.019 (*P* < 0.05). The area under ROC curve (AUC) of DKD identified by the above indicators was 0.914, 0.643, 0.807, and 0.797, respectively. The AUC of Joint prediction probability of DKD recognition was 0.914, and the sensitivity and specificity of DKD diagnosis were 1.0 and 0.806, respectively. (Take ≥0.251 as the diagnostic cutoff point).

**Conclusion:**

LncRNA Malat1 is highly expressed in DKD patients, and the combined detection of ACR, creatinine, *α*1-MG, and LncRNA Malat1 with diabetes mellitus may be the best way to diagnose diabetic nephropathy.

## 1. Introduction

Diabetic kidney disease (DKD) has become the primary etiology for chronic kidney disease in China. The morbidity and mortality associated with DKD has been rapidly increased over the past 20 years [1, 2]. According to the latest epidemiological data, DKD is considered as the primary cause for the progression to dialytic end-stage kidney diseases in the urban population [3–5]. Long noncoding RNA (lncRNA) plays an important role in the modulation of various cellular responses, system development, and pathogenesis [6]. Recent studies have confirmed the involvement of lncRNA in DKD processes such as renal fibrosis, extracellular matrix deposition, inflammatory response, oxidative stress, and apoptosis of renal tubular epithelial cells [7]. It played a vital role in DKD pathogenesis and progression via a series of mechanisms. MALAT1, also known as noncoding nuclear-enriched abundant transcript 2 (NEAT2), was initially detected in the year 2003 by Ji et al. [8] in nonsmall cell lung cancer (NSCLC). It is regarded as one of the highly conservative lncRNAs in mammals. The latest studies have reported MALAT1 as one of the first lncRNAs detected and was significantly upregulated under high-glucose culture in retinal endothelial cells and in the retina of diabetic mice model [9, 10]. LncRNA MALAT1 modulates inflammatory response in diabetes-initiated microvascular complications, such as DKD and diabetic retinopathy [11–13]. Therefore, this study mainly aimed to explore the role of lncRNA MALAT1 in DKD and its potential pathogenesis, which could benefit from the identification of reliable biomarkers and novel therapeutic targets.

## 2. Materials and Methods

### 2.1. Subjects

This study enrolled 47 diabetic patients who sought medical advice at the Affiliated Hospital, Youjiang Medical College for Nationalities between January 2017 and December 2017, which included 20 cases with type 2 diabetes mellitus (T2DM) (12 males and 8 females, with a mean age of 54.85 ± 14.24 years) and 27 cases were with DKD (16 males and 11 females, with a mean age of 56.3 ± 12.67 years). Meanwhile, 14 nondiabetic healthy volunteers were enrolled as control group who checked into our health examination center (8 males and 6 females, with a mean age of 49.57 ± 12.54 years). No statistical difference was detected in gender and age between DM, DKD, and control groups.

DKD patients should meet the enrollment criteria of DKD diagnosis standards of China T2DM prevention and treatment guidelines 2019 [14]. T2DM patients (1) who meet the WHO diabetes mellitus diagnosis standards 1999; and (2) without other severe diseases that affect glycemia or albuminuria, such as pregnancy, infection, contagious disease, malignancy, and drug medication. Subjects in control group were enrolled by referring to the above 2 conditions, but missing diabetes mellitus diagnostic standards by glucose tolerance test.

Patients in DM and DKD groups were given insulin glargine (Sanofi-Aventis Deutschland Gmb H) combined with acarbose (Hangzhou Huadong Medicine Co., Ltd., China) to control blood glucose. In order to reduce the experimental error, the enrolled patients should be with only DKD; those with cardiovascular and cerebrovascular diseases were excluded.

This study was reviewed and approved by our hospital ethics committee and was conducted after gaining full informed consent from patients.

### 2.2. Anthropometrical and Biochemical Parameter Measurements

Study Subjects. General data were collected from all study subjects, which included peripheral blood sample, urine sample, body height, body weight, blood pressure, and heart rate. Also, body mass index (BMI) was calculated using the formula: BMI = body weight/body height2 (kg/m2). Mindray Automatic Biochemical Analyzer BS-2000M (Shenzhen, China) was used for measuring biochemical indexes of superoxide dismutase (SOD), creatinine (Cr), glycated hemoglobin (HbA1c), and blood lipids. Insulin was measured with magnetic particle chemiluminescence instrument (Autobio). Specific Proteins Analyzer BA400 (Bio Systems, Spain) was used for detecting ACR, urine *β*2-MG, and urine *α*1-MG. All these parameters were measured by our technicians who received specific training. SI units were applied accordingly.

Measurement of urine *β*2-MG, *α*1-MG, microalbumin (UMA) and creatinine, and ACR calculation.

Fasting midstream urine sample of 5 mL was collected and centrifuged at 3000 r/min for 10 min to collect the supernatant. This was followed by immunoturbidimetry analysis of UMA and urine Cr using Specific Proteins Analyzer BA400. Next, ACR was calculated using the formula: ACR = urine albumin/creatinine. ACR readout <3 mg/g was defined as normal while ≥3 mg/g abnormal; urine *β*2-MG readout <0.3 mg/L was normal while ≥0.3 mg/L abnormal; urine *α*1-MG readout <30 mg/L was normal while ≥30 mg/L abnormal. The normal range of blood creatinine was 50~106 *μ*mol/L.

### 2.3. Real-Time PCR Analysis of LncRNA MALAT1 Expression

#### 2.3.1. Total RNA Extraction and Reverse Transcription

5 mL venous blood was drawn from all patients and added to 9 mL of red cell lysis buffer, followed by mixing before proceeding to centrifugation at 2500 rpm for 5 minutes at 4°C in an ultralow temperature centrifuge. The supernatant was then discarded, and the extracted leukocytes were rinsed with 1 mL PBS buffer before preservation at -80°C in a freezer. The total RNA of leukocytes was extracted using Trizol kit (Pufei Bio, Shanghai, China), which was then proceeded to reverse transcription according to Promega M-MLV kit (RiboBio, Guangzhou, China) manual. The reverse transcription primers were also purchased from RiboBio. All the above procedures were strictly conducted with reference to the kit manual.

#### 2.3.2. Quantitative Real-Time PCR (qRT-PCR)

PCR analysis was performed using SYBR Green (TIANGEN Bio, Beijing, China). PCR reaction system was as follows: 95°C for 15 min, 95°C for 10s, 60°C for 20s; 40 cycles. PCR reaction was evaluated with a melting curve. Each sample was performed in triplicate, and the average value was taken for analysis. LncRNA MALAT1 expression level was reflected by 2^-*ΔΔ*Ct^ value, which was calculated using the formula: ΔΔCt = (CTMALAT1 − CTGADPH)DM − (CTMALAT1 − CTGADPH)con. The expression level of GADPH genes is the best reference to evaluate relative changes in gene activity in diabetic/high glucose exposed glomerular tissues [15].

Primer sequences were shown in Table 1.

### 2.4. Statistical Methods

Variance analysis was conducted using SPSS 25.0 software and expressed as *x* ± *s*. Chi-square test was used to analyze the relations of LncRNA Malat1 with each clinicopathological parameter. Pearson's linear regression method was applied to analyze the correlation of LncRNA Malat1 with proteinuria, glomerular filtration rate, and other clinicopathological features. In order to explore the risk factors of DKD in patients with diabetes mellitus, the indexes with difference were included in the binary regression analysis, then drew the ROC curve of each factor in identifying DKD, and jointed multifactor ROC curve to calculate the area under the ROC curve (AUC), and analyzed the diagnostic cross-section points of each index and the sensitivity and specificity of DKD identification; *P* < 0.05 was considered to be statistically significant.

## 3. Results

### 3.1. Comparisons of General Clinical Data in Three Groups

All the three groups showed no statistical differences in either gender, age, disease duration, BMI, ACR, urine *β*2-MG, or triglycerides (*P* > 0.05), while statistical significance was observed in parameters such as urine *α*1-MG, CR, EGFR, SOD, FBG, and HbA1C (*P* < 0.05), shown in Table 2.

### 3.2. Differential Expression of LncRNA MALAT1 in T2DM, DKD, and Healthy Control Groups

**Figure 1:**
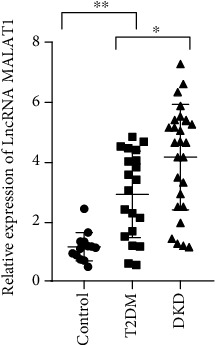
Expression of circulating LncRNA MALAT1 in T2DM patients, DKD patients, and healthy controls.

Compared with healthy control group, LncRNA MALAT1 expression was shown to be obviously upregulated in T2DM and DKD patients, with statistical significance (*F* = 20.24, *P* < 0.001, Figure1). Further analysis uncovered the marked rise of LncRNA MALAT1 expression in DKD patients over T2DM patients, showing statistical significance (*F* = 0.72, *P* < 0.05, Figure1).

### 3.3. LncRNA MALAT1 Expression in Injured Renal Tubules

**Figure 2:**
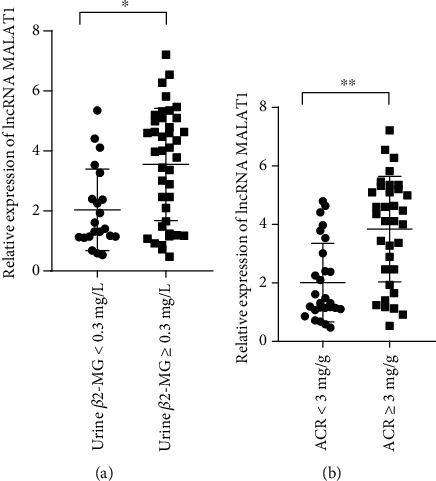
(a) Association of LncRNA MALAT1 levels with the degree of urine *β*2. (b) Association of LncRNA MALAT1 levels with the degree of ACR.

The results of lncRNA MALAT1 expression in renal tubule urine *β*2-MG revealed obvious upregulation of serum lncRNA MALAT1 in DM patients with abnormal *β*2-MG when compared to those with normal urine *β*2-MG, showing statistically significant difference (*t* = 3.62, *P* < 0.05, Figure 2(a)).

The results of lncRNA MALAT1 expression in ACR indicated upregulation of serum lncRNA MALAT1 in DM patients with abnormal ACR over those with normal ACR, showing statistically significant difference (*t* = 4.38, *P* < 0.001, Figure 2(b)).

### 3.4. Correlation of lncRNA MALAT1 Expression in DM Patients with SOD, ACR, Urine *β*2-MG, Urine *α*1-MG, Cr, and HbA1c

**Figure 3:**
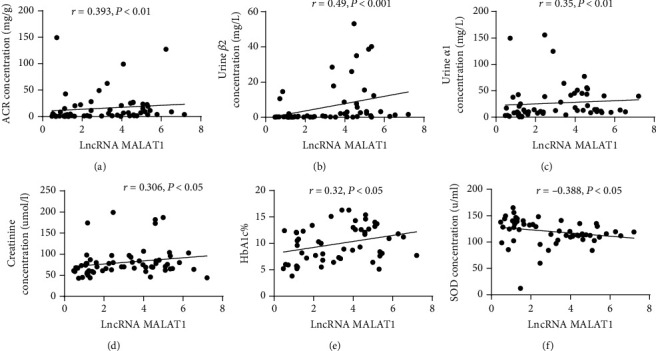
Correlation analysis of the expression LncRNA MALAT1 with the concentration of ACR, urine *β*-MG, urine *α*1-MG, creatinine, HbA1c, and SOD.

Pearson's linear correlation coefficient was used to analyze the correlation of lncRNA MALAT1 expression in DM patients with SOD, ACR, urine *β*2-MG, urine *α*1-MG, Cr, and HbA1c. The results revealed that lncRNA MALAT1 expression was positively correlated with ACR, urine *β*2-MG, urine *α*1-MG, Cr, and HbA1c (Figures 3(a)–3(e)), while a negative correlation was observed with SOD (Figure 3(f)).

### 3.5. Value of Joint Indicators and Joint Prediction Probability in Identifying DKD

Binary regression analysis showed that ACR, creatinine, *α*1-MG, and LncRNA Malat1 (2^-*ΔΔ*Ct^) were the risk factors of DKD, with OR value of 1.166, 1.031, 1.031, and 2.019 (*P* < 0.05). The area under ROC curve (AUC) of DKD identified by the above indicators were 0.914, 0.643, 0.807, and 0.797, respectively. The AUC of Joint prediction probability of DKD recognition was 0.914, the sensitivity and specificity of DKD diagnosis were 1.0 and 0.806, respectively. (Take ≥0.251 as the diagnostic cutoff point), shown in Table 3.

**Figure 4:**
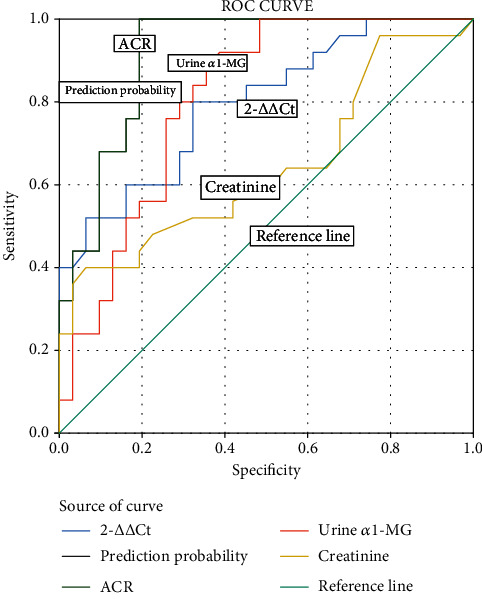
ROC curve analysis of indicators in identifying DKD in patients with diabetes mellitus.

Draw the ROC curve of each index in identifying DKD, the area under the ROC curve (AUC) was calculated by multiparameter analysis and analyzed the diagnostic cross-section points of each index and the sensitivity and specificity of DKD identification (Figure 4).

## 4. Discussion

DM is a chronic metabolic disease with its complications affecting the kidney, retina, and nerve. The number of global DM patients reaches to about 366 million by 2030 and 592 million by 2035, which is 1/10 of the earth's population [16, 17]. DKD is a common DM complication that lasts long and is poorly controlled [18–20]. In China, about 20-40% of DM patients have DKD, and it has become the primary cause of CKD and end-stage kidney diseases [3, 21]. Despite clear understanding of the molecular mechanism of DKD, increasing evidences demonstrated the critical role of genetic regulation in its pathogenesis. More and more evidences reported the key role of lncRNA in diverse diseases [22]. Previous studies have confirmed MALAT1 dysregulation in DKD progression, and it has a value of being a diagnostic target.

This study found significant upregulation of lncRNA MALAT1 in T2DM and DKD patients when compared to healthy controls, and this was in agreement with the study results of Zhang et al. [23]. DKD deteriorates slowly and is regarded as the main early manifestation of kidney enlargement and rise in the glomerular filtration rate (GFR), without any clinical symptoms [24–27]. Along with disease progression, GFR increase is coupled with change in the filtration membrane charges and occurrence of urine microalbumin (mAlb). But GFR still appeared to be within normal range, with no relevant emergence of clinical symptoms. The sole abnormality involves urine mAlb. So far, mAlb is an important index for laboratory diagnosis of early DKD. In clinical practice, ACR is adopted to correct the fluctuations of random urine mAlb levels. In our study, the kidney function status of DKD and DM patients was evaluated by ACR, urine *β*2-MG, urine *α*1-MG, and Cr. The relative lncRNA MALAT1 expression was increased in DM patients with high ACR, showing a significant positive correlation. Similarly, the relative lncRNA MALAT1 expression was also increased in DM patients with high urine *β*2-MG, showing a significant positive correlation. In this study, the lncRNA MALAT1 expression showed a positive correlation with urine *α*1-MG, Cr, and HbA1c, implying lncRNA MALAT1 involvement in kidney impairment. The study conducted by Li et al. [28]reported the role of MALAT1, miR-23c, and its target gene pyrophosphorylation-related protein ELAVL1 in renal tubular epithelial cells, and these played a potential role by affecting pyrophosphorylation cell death signaling pathway in DKD. LncRNA MALAT1 expression was upregulated in hyperglycemic conditions. Luciferase assay confirmed MALAT1 as the target gene of miR-23c. Therefore, downregulation of cellular miR-23c increased the expression of pyrophosphorylation-related protein ELAVL1 and NLRP3, followed by Caspase-1 activation, and subsequent secretion of proinflammatory chemokines IL-1*β* and IL-18, thereby promoting inflammatory response. In contrast, MALAT1 downregulation or miR-23c upregulation elicited suppression of ELAVL1 expression, which inhibited pyrophosphorylation signaling pathway, thereby modulating DKD initiation and development.

Our study also uncovered the negative correlation of lncRNA MALAT1 expression with SOD. Bulky production of activated oxygen and overwhelming oxidative stress response were considered as important pathological changes during DKD development [29]. SOD, an antioxidative enzyme, catalyzes disproportionation of superoxide anion radical to hydrogen peroxide and oxygen, maintaining the balance of free radicals in the body [30]. Previous reports demonstrated decreased SOD level in DM patients, which increased the peroxides in the glomerular microvascular system, which subsequently escalated the cytotoxic effect caused by peroxides, thereby resulting in DM renal cell damage [31]. SOD level was negatively correlated with the severity of renal function impairment, indicating the importance of SOD during various stages of DKD development. SOD is regarded as a sensitive index for dynamic monitoring of DKD development.

Binary regression analysis showed that ACR, creatinine, *α*1-MG, and LncRNA Malat1 (2^-*ΔΔ*Ct^) were the risk factors of DKD, the sensitivity and specificity of DKD can be further improved to 100% and 80.6% by using the combined predictor variables, which is higher than that of single index. This study initially reported high expression of lncRNA MALAT1 in the circulating of DKD patients and showed correlation with ACR, urine *β*2-MG, urine *α*1-MG, and Cr. This study would further benefit from the theories of DKD pathogenesis and provide novel targets for DKD gene-targeted therapy. Also, our findings provide references to seek out biomarkers for diagnosis and outcome prediction of DKD and offer a novel strategy for managing clinically intractable DKD. Nonetheless, the influential factors of DKD are diverse. The specific pathogenesis, diagnosis, and outcome assessment should be further elucidated.

The inadequacies of this article are (1) insufficient sample size. (2) Long time span of sample collection, causing some RNA to degrade, Malat1 has decreased in the diabetic nephropathy group. (3) Since other nephropathy except diabetic nephropathies has been excluded in this study, this model is only applicable to the patients with diabetes mellitus history and who have been evaluated and considered as diabetic nephropathies.

## Figures and Tables

**Table 1:** LncRNA MALAT1 and GADPH gene primer sequence.

Primer	Sequence
MALAT1 upstream	5′- CAGACCACCACAGGTTTACAG-3′
MALAT1 downstream	5′-AGACCATCCCAAAATGCTTCA-3′
GADPH upstream	5′-TGACTTCAACAGCGACACCCA-3′
GADPH downstream	5′-CACCCTGTTGCTGTAGCCAAA-3′

**Table 2:** Comparisons of general clinical data in three groups.

	Healthy control group (*N* = 14)	T2DM groups (*N* = 20)	DKD groups (*N* = 27)	*F* or *χ*^2^ value	*P* value
Sex	Male 8	Male 12	Male 16	0.29	0.986
	Female 61	Female 8	Female 11		
Age (years)	49.57 ± 12.54	54.85 ± 14.24	56.3 ± 12.67	1.228	0.3
Disease duration (years)	0	4.65 ± 3.86	6.0 ± 3.67		0.23
FBG (mmol/L)	4.06 ± 0.66	7.41 ± 2.33	8.56 ± 4.0	10.41	0.00^∗∗^
SOD (*μ*/ml)	139.15 ± 18.2	116.32 ± 28.03	112.77 ± 18.21	7.09	0.002^∗^
BMI (kg/m2)	24.02 ± 5.89	25.25 ± 4.54	24.35 ± 4.26	0.326	0.72
ACR (mg/g)	1.046 ± 2.20	5.25 ± 7.71	24.81 ± 29.81	8.31	0.001∗
Urine *β*2-MG (mg/L)	2.0 ± 4.56	6.61 ± 13.7	7.01 ± 12.13	0.963	0.39
Urine *α*1-MG (mg/L)	7.31 ± 9.41	19.16 ± 18.92	36.62 ± 34.47	6.47	0.003^∗^
Cr (*μ*mol/L)	65 ± 17.1	72.2 ± 14.15	94.78 ± 45.49	4.84	0.011∗
TG (mmol/L)	2.05 ± 0.28	2.25 ± 1.67	3.06 ± 4.29	0.60	0.551
LDL (mmol/L)	2.27 ± 0.45	2.75 ± 1.02	2.83 ± 1.04	1.77	0.180
HbA1C (%)	5.26 ± 0.48	10.42 ± 2.81	10.81 ± 3.11	22.60	0.000^∗∗^
EGFR (ml/min)	111 ± 48.11	95.85 ± 35.34	74.44 ± 38.6	4.20	0.020^∗^

*P* < 0.05 indicated statistical significance, ^∗^*P* < 0.05, ^∗∗^*P* < 0.001. Abbreviations: SOD: superoxide dismutase; BMI: body mass index; HbA1c: glycated hemoglobin; FBG: fasting blood-glucose; TG: triglyceride; LDL-C: low-density lipoprotein cholesterol; Cr: creatinine; eGFR: estimated glomerular filtration rate; ACR: urine albumin/creatinine; *β*2-MG: urine *β*2-microglobulin; *α*1-MG: urine *α*1-microglobulin (*α*1-MG).

**Table 3:** Diagnostic value of indicators in identifying DKD in patients with DM patients.

Detection variable	AUC	Diagnostic cutoff point	Sensitivity	Specificity
Prediction	0.914	0.251	1	0.806
Probability				
2^-*ΔΔ*Ct^	0.79	2.429	0.8	0.677
ACR	0.914	2.765	1	0.806
Creatinine	0.643	9.2	0.4	0.935
Urine *α*1-MG	0.807	9.9	0.92	0.613

## Data Availability

The data for the current study are available from the corresponding author upon reasonable request.
